# Investigation of Intertriginous Mycotic and Pseudomycotic (Erythrasma) Infections and Their Causative Agents with Emphasize on Clinical Presentations

**Published:** 2018-09

**Authors:** Samaneh HALVAEE, Roshanak DAIE GHAZVINI, Seyed Jamal HASHEMI, Ensieh ZIBAFAR, Saeed YEKANINEJAD, Mohsen GERAMISHOAR, Heidar BAKHSHI, Shahram MAHMOUDI, Hasti KAMALI SARVESTANI, Razieh YAGHOUBI, Leila HOSSEINPOUR, Zeinab BORJIAN

**Affiliations:** 1. Dept. of Medical Parasitology and Mycology, School of Public Health, Tehran University of Medical Sciences, Tehran, Iran; 2. Dept. of Epidemiology and Biostatistics, School of Public Health, Tehran University of Medical Sciences, Tehran, Iran; 3. Students’ Scientific Research Center, Tehran University of Medical Sciences, Tehran, Iran

**Keywords:** Intertrigo, Tinea, Candidiasis, Erythrasma, Signs and symptoms

## Abstract

**Background:**

Intertrigo is an erythematous inflammatory condition with multiple etiologies including fungi and bacteria. Intertrigo manifests in different clinical forms with various complaints. This study was conducted to evaluate the causative agents of intertriginous infections with emphasize on clinical presentations.

**Methods:**

This descriptive cross-sectional study was carried out in 2015–2016, on 188 patients with clinical suspicion of superficial and cutaneous intertriginous infections in Tehran, Iran. Demographic and additional related data were obtained by questionnaire from all participants. Specimens were collected by gentle scraping of the affected areas. Direct examination and culture were performed for all specimens and grown colonies were identified based on the macroscopic and microscopic features. Supplementary tests were done whenever needed. Data were analyzed in SPSS.

**Results:**

Overall, 80 (42.5%) cases with the mean age of 43.5 yr were confirmed for intertrigo. Dermatophytosis was the predominant cause in this study with 36 (45%) cases followed by erythrasma (28 cases, 35%), tinea versicolor (10 cases, 12.5%) and candidiasis (6 cases, 7.5%). Intertrigo lesions with dermatophytic agents significantly were observed in groin in comparison to different infections among body sites (*P*<0.05). Itching was the most common clinical presentation (57 cases, 71.3%) and also significant association between different infections and clinical manifestations were observed (*P*<0.05).

**Conclusion:**

Different clinical manifestations may be observed in infectious intertrigo. Regarding the significant association observed in this study, some clinical features can be used for presumptive diagnosis of diseases but further studies are required to make it clear.

## Introduction

Intertrigo or skin fold dermatitis is an erythematous inflammatory condition with multiple etiologies which is one of the most common complaints of patients in dermatology clinics. This condition could involve both large and small body folds such as groin, inframammary and axillary folds and interdigital spaces (toe and finger webs). There are various predisposing factors with local or systemic nature such as moisture, heat, obesity, skin friction, lack of proper ventilation, poor hygiene, diabetes, allergy, immunosuppression states and drug consumption (1–4).

Intertrigo has a spectrum of infectious and non-infectious causes (5). Skin fungal infections are among the major causes of intertrigo and affect a large scale of people worldwide (6). Dermatophytosis is a common fungal infection refers to a set of cutaneous diseases divided to eight groups based on the anatomical sites of the body and cause by species from three genera including *Trichophyton*, *Epidermophyton* and *Microsporum* (7). Other common superficial fungal infections of skin folds can be classified as candidiasis and tinea versicolor which are due to *Candida* species and *Malassezia* species, respectively (8).

Erythrasma is another infectious intertrigo due to *Corynebacterium minutissimum*. Although this pathogen is a bacterial organism, it usually accompanies with other bacteria, dermatophytes, and yeasts (9). Though, this infection needs to be considered along with other fungal intertrigo infections. There are various skin disorders affecting the intertriginous areas which resemble in clinical appearances. Though, it is of importance to distinguish among various etiologies. Direct examination and culture are confirmatory tests for diagnosis of fungal skin infections which usually are overlooked in clinical practice and diagnosis are mainly based on the clinical manifestations (4, 10). Therefore, application of these tests for accurate identification of intertriginous infections could provide valuable epidemiologic data. Furthermore, analysis of clinical features of confirmed infections may provide associations between specific presentations and diseases which could be beneficial for situations which a mycology laboratory is not available.

Thus, this study was carried out with the aim to diagnose the intertriginous mycotic (dermatophytosis, candidiasis, and tinea versicolor) and pseudomycotic (erythrasma) infections among suspected patients along with identification of their etiologies with emphasize on clinical features of lesions.

## Materials and Methods

### Patients

This descriptive cross-sectional study was carried out in a two year period (2015–2016) on 188 patients with clinical suspicion of superficial and cutaneous intertriginous infections and referred to the Medical Mycology Laboratory of School of Public Health, Tehran University of Medical Sciences, Tehran, Iran.

### Questionnaire preparation and ethical considerations

A questionnaire covering demographic data, history of fungal infections, underlying diseases, clinical features of lesions such as scaling and inflammation and the site of lesions on body was prepared and filled for all the patients.

Since all participants were referred to the Medical Mycology Laboratory to their own discretion, so there was no need to get approval from the ethics committee.

### Specimen collection

Samples were collected by gentle scraping of the affected areas (groin, axillary inframammary, abdominal, perianal folds, and interdigital spaces) and were kept in sterile petri dishes for further examinations.

### Microscopic examination and culture

Skin scrapings were divided into two parts. One was used for direct microscopic examination using 10% potassium hydroxide (KOH) and methylene blue staining. The other part was cultured on sabouraud dextrose agar (Merck, Germany) containing chloramphenicol (50 mg/l, Sigma) with or without cycloheximide (500 mg/l, Sigma) and incubated at 28 °C for four weeks. Cultures were checked daily.

### Identification of fungal species

Fungal colonies were examined macroscopically for morphology, texture, and surface and reverse pigmentation. Microscopic examination of colonies was done using lactophenol cotton blue wet mount preparation of teased mounts and slide cultures. Supplementary tests were performed whenever needed. For instance, urea hydrolysis test was done to distinguish between *T. rubrum* and *T. mentagrophytes*.

### Statistical analysis

The statistical analysis was performed using SPSS ver. 21.0 for Windows and the data were expressed as means±SD and percentile of the total. Chi-square test was used to compare frequency of features in different intertriginous skin infections with *P*<0.05 considered significant.

## Results

Among 188 patients including 47 (58.75 %) males and 33 (41.25 %) females, intertriginous skin infections were confirmed in 80 cases (42.5%) with the mean age of 43 (age range: 4 – 77 yr). The age range of 41–51 was the most commonly affected age group.

The causative agents of all fungal infections were identified. Dermatophytosis was the most common infection observed in 36 patients (45%) and *T. rubrum* was the predominant etiology. Erythrasma was the second most common infection confirmed in 28 patients (35%) and the etiology of all cases were considered *C. minutissimum.* Tinea versicolor and candidiasis were observed in 10 (12.5%) and 6 (7.5%) cases, respectively (Table 1).

**Table 1:** The frequency of different intertriginous skin disorders and causative agents among 188 patients included in this study

***Clinical form***	***Causative agent***	***Frequency (%)***
Dermatophytosis		36 (45%)
	*Trichophyton rubrum*	12
	*Epidermophyton floccosum*	10
	*Trichophyton mentagrophytes*	8
	*Trichophyton tonsurans*	2
	*Trichophyton verrucosum*	2
	*Microsporum canis*	1
	*Microsporum gypseum*	1
Erythrasma		28 (35%)
	*Corynebacterium minutissimum*	28
Tinea versicolor		10 (12.5%)
	*Malassezia* spp.	10
Candidiasis		6 (7.5%)
	*Candida albicans*	4
	*Candida parapsilosis*	2
Total		80 (100%)

The distribution of various intertriginous infections based on the body sites was also recorded (Table 2). Generally, groin was the most common site followed by interdigital spaces, axillary, inframammary folds and neck. There was a statistically significant difference between the distribution of various intertriginous infections among different body sites (*P*=0.001).

**Table 2:** The frequency of different intertriginous skin disorders based on various body sites

***Body sites***	***Intertriginous skin disorders***				***P***
	***Dermatophytosis***	***Erythrasma***	***Tinea versicolor***	***Candidiasis***	
Groin	15	10	4	4	0.001
Toe web spaces	21	16	0	1	
Axillary folds	0	2	4	0	
Inframammary folds	0	0	1	1	
Neck	0	0	1	0	

In total seven types of clinical features were recorded for intertriginous infections. Itching was the most common clinical finding observed in 57 of 80 patients followed by scaling in 44 patients. The lowest frequency was noted for geographic skin rashes observed only in three patients. According to statistical analysis, there were significant associations between various intertriginous infections and the type of clinical features. Table 3 represents the frequency of various clinical features of skin lesions among different infections. Moreover, detailed data clinical findings of intertriginous infections based on various body sites are shown in Table 4.

**Table 3:** The frequency of different clinical findings in various intertriginous skin disorders

**Clinical findings**	**Skin disorders**				*P*
	***Tinea versicolor***	***Dermatophytosis***	***Erythrasma***	***Candidiasis***	
	***(N=10)***	***(N=36)***	***(N=28)***	***(N=6)***	
Itching	6(60.0%)	33(91.7%)	13(46.4%)	5(83.3%)	0.001
Scaling	1(10.0%)	35(97.2%)	3(10.7%)	5(83.3%)	<0.001
Pigmentation	10(10.0%)	0	27(96.4%)	0	<0.001
Satellite lesions	1(10.0%)	0	0	3(50.0%)	<0.001
Geographic skin rash	3(30.0%)	0	0	0	<0.001
Marginated skin lesions	0	9(25%)	0	0	0.006
Inflammation	0	22(61.1%)	0	2(33.3%)	<0.001

**Table 4:** The frequency of various clinical findings in different intertriginous skin infections based on the body sites

***Intertriginous skin disorder***	***Body site***	***Clinical findings***					
		***Itching***	***Scaling***	***Erythema***	***Satellite lesions***	***Inflammation***	***Marginated skin lesions***
Dermatophytosis	groin	20	15	0	0	12	9
	Toe web spaces	13	21	0	0	10	0
Candidiasis	groin	2	4	0	2	1	0
	Toe web spaces	1	1	0	0	0	0
	Inframammary folds	2	1	0	1	1	0
Tinea versicolor	groin	3	0	4	0	0	0
	Inframammary folds	1	0	2	1	0	0
Erythrasma	groin	6	2	9	0	0	0
	Toe web spaces	3	1	9	0	0	0

**Fig. 1:**
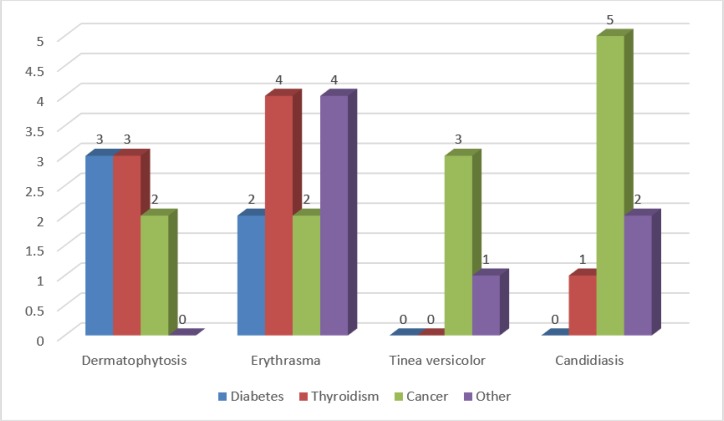
The frequency of different underlying diseases in various intertriginous skin disorders (data labels are the number of patients in each group)

Based on the data recorded by the questionnaire, some underlying diseases such as diabetes, thyroidism, and cancer were reported by some patients (Fig. 1). Meanwhile, there was no statistically significant association between these underlying diseases and the occurrence of intertriginous infections (*P*=0.245).

## Discussion

In this study, prevalence of intertrigo and erythrasma was 43%. This finding is not in accordance with the result that reported the prevalence of intertrigo in hospitalized patients and nurses was 6% and 17%, respectively (3). There is not any appropriate pattern for intertrigo prevalence. It could be due to neglecting of these diseases.

In different sites of body with more rubbing, wet and less ventilation fungal diseases were seen more. These reports confirmed our results (11–14). Clinical manifestations were various in different geographic regions due to climatic factors (8).

According to our findings, the highest prevalence of erythrasma and cutaneous fungal infections was recorded in the age group of 41–51 yr (21.2%). The most prevalence of superficial and cutaneous diseases was reported in 60–70 and 20–30 yr old, respectively. These variations could be mainly due to different patterns of age grouping or differences in geographical regions (15).

In the present study, erythrasma and intertrigo fungal infections often tend to appear in toe web spaces and then in groin rather than the other sites. This finding could be due to their specific physiological and anatomical characterizations which are inconsistent with reported results (16). In addition, the prevalence of dermatophyte infections in patients without clinical symptoms (hidden forms) is similar to patients with clinical signs. Therefore, they can be thought of as reservoirs of disease.

In this study, *T. rubrum* with 12 cases (33.3%) and *T. mentagrophytes* with 8 cases (22.2%) were identified as the most common dermatophyte in toe web spaces and *E. floccosum* with 10 cases (27.7%) was the most one in groin, that from this point of view, other studies were in accordance with our results (17–19).

Being marginalized in all superficial and cutaneous *candida* infections in 6 cases (100%) was the most common symptom in our study. The other symptoms were crusting, 35 cases (79.5%) and then itching 33 cases (57.9%). This finding was also similar to other studies (20–23).

According to our findings, history of family members to dermatophytosis was founded as the most common predisposing factor that was also in accordance with other studies (24).

This study indicated that persons with moderate body mass index (BMI) had more mycotic and pseudomycotic (erythrasma) infections, while this finding is inconsistent with others results that informed, persons with high BMI (>30) have more prevalence of intertrigo infections (25, 26).

## Conclusion

Overall, crusting, itching, inflammation, marginality, lack of pigmentation and satellite lesions can be suspected to be dermatophytosis.

Moreover, cutaneous lesions with symptoms such as inflammation, satellite lesions, itching, non-pigmentation, crusting and marginality suspected to be candidiasis.

Pigmented lesions with limited itching and lack of crusting, marginality, satellite lesions and inflammation could be a lead to tinea versicolor and skin lesions without any inflammation, itching, marginality, satellite lesions with pigmentation (red to brown) could be diagnosis key for erythrasma.

## Ethical considerations

Ethical issues (Including plagiarism, informed consent, misconduct, data fabrication and/or falsification, double publication and/or submission, redundancy, etc.) have been completely observed by the authors.
